# Clinical and Biological Evaluation of Chemo-Mechanical Caries Excavation with Brix 3000 in Primary Molars: An 18-Month Prospective Study

**DOI:** 10.3390/medicina62040615

**Published:** 2026-03-24

**Authors:** Zornitsa Lazarova, Nadezhda Mitova

**Affiliations:** Department of Pediatric Dentistry, Faculty of Dental Medicine, Medical University—Sofia, 1 Georgi Sofiyski St., 1431 Sofia, Bulgaria; n.mitova@fdm.mu-sofia.bg

**Keywords:** Brix 3000, dental caries removal, dental restoration failure, minimally invasive surgical procedures, pediatric dentistry, tooth, deciduous

## Abstract

*Background and Objectives*: Caries in primary teeth are characterized by rapid and often asymptomatic progression, with early dentin involvement and potential extension to the pulp. Untreated lesions may lead to complications that affect the development of the permanent dentition. The aim of this prospective study was to evaluate the clinical and biological effectiveness of chemo-mechanical controlled caries excavation using Brix 3000 compared to conventional treatment in primary molars over an 18-month follow-up period. *Materials and Methods*: A total of 82 children aged 4–7 years were included, each presenting with at least one carious lesion in a primary molar classified as International Caries Detection and Assessment System (ICDAS II) code 05 or 06. The carious lesions were divided into two groups according to the method of excavation: Group 1 (control), which contained 40 lesions treated with conventional bur excavation, and Group 2, which contained 42 lesions treated with chemo-mechanical excavation using Brix 3000. In all cases, excavation was controlled using a fluorescence-based device (ProFace). Clinical performance was evaluated using an assessment protocol adapted from the FDI (Fédération Dentaire Internationale) clinical criteria for the evaluation of direct and indirect restorations, with particular emphasis on biological outcomes. Follow-up examinations were performed after 1 week and 1, 3, 6, 12, and 18 months, and included radiographic evaluations. *Results*: After 18 months, chemo-mechanical caries excavation with Brix 3000 demonstrated a biological success rate of 100%, with no reported acute symptoms or complications. Esthetic criteria showed a success rate of 65% at 18 months, while anatomical and functional criteria demonstrated success rates of 95% and 98%, respectively. In the conventional bur excavation group, biological success reached 100%, while the esthetic, anatomical, and functional success rates were 61.3%, 93.5%, and 100%, respectively. No significant differences were observed between groups (*p* > 0.05). *Conclusions*: Chemo-mechanical controlled caries excavation using Brix 3000 represents a clinically effective and biologically reliable alternative to conventional caries excavation for the treatment of carious lesions in primary molars.

## 1. Introduction

Caries in primary teeth is one of the most common diseases in pediatric dentistry and remains a significant global health concern [1,2,3]. The prevalence of caries in primary teeth is associated with their anatomical and physiological characteristics, children’s dietary habits, and oral hygiene practices [2]. Preserving primary teeth until their physiological exfoliation is crucial for proper development of the masticatory system, maintaining space for permanent teeth, and the overall health of the child [4,5].

Untreated carious lesions in the primary dentition can lead to a range of complications, including the development of pulpitis and periodontitis, spontaneous and nocturnal pain, feeding difficulties, and impaired quality of life for children. Moreover, premature loss of primary teeth may result in occlusal disturbances and an increased risk of caries in permanent teeth [5,6,7]. For these reasons, early and appropriate management of carious lesions in primary teeth is of paramount importance [4,6,8].

Currently, more conservative treatment methods can be applied to primary teeth in accordance with the principles of minimally invasive dentistry. The main principle of minimally invasive treatment is the maximal preservation of healthy tooth structures while only removing irreversibly damaged tissue. This approach is particularly relevant in pediatric dentistry as it reduces pain and fear and protects the pulp from unnecessary exposure during cavity preparation [9,10,11]. Chemo-mechanical caries excavation, involving the selective removal of carious dentin, represents one such method [12].

Brix 3000 is an enzymatic agent based on the papain enzyme, which can be used for chemo-mechanical excavation of carious lesions in primary teeth. Papain is bio-encapsulated using proprietary EVE (Encapsulated Vesicular Enzyme) technology, which stabilizes the enzyme and enhances its activity. The selective enzymatic removal of infected dentin relies on the absence of the anti-protease alpha-1-antitrypsin in carious dentin, which normally inhibits collagen proteolysis, thereby enabling papain activity [13,14,15]. These characteristics make Brix 3000 a viable alternative to conventional bur excavation, which can be associated with an unpleasant experience for children due to vibration and noise during treatment [15].

Recent clinical studies and systematic reviews have reported clinical success rates comparable to those of conventional cavity preparation, with significantly reduced discomfort and anxiety in pediatric patients [13,16]. Moreover, enzymatic agents such as Brix 3000 have demonstrated favorable outcomes in terms of pulp preservation and improved patient cooperation during treatment. Systematic reviews further indicate that papain-based chemo-mechanical methods reduce acoustic and nociceptive stimuli and improve procedural tolerance compared with conventional rotary excavation [16]. Clinical data describe Brix 3000 as a minimally invasive agent that facilitates selective dentin removal and enhanced patient comfort [17]. In addition, broader discussions of papain-based gels have highlighted reduced patient anxiety and lower procedural invasiveness, while recent clinical evaluations report a performance comparable to conventional bur excavation with favorable patient experiences [17,18]

Despite the proven advantages of enzymatic agents, evaluating their clinical efficacy requires systematic follow-up of treated cases over time. Prospective observation allows for an objective assessment of biological, esthetic, anatomical, and functional outcomes, as well as the early detection of potential complications. Therefore, monitoring clinical outcomes is a critical component in evaluating the effectiveness and safety of new and alternative treatment approaches in pediatric dentistry [19].

Although Brix 3000 has been introduced as an enzymatic agent for chemo-mechanical caries removal and has been evaluated in several clinical and laboratory studies, the available evidence remains limited and fragmented. Most published reports focus on immediate clinical performance, handling properties, or patient comfort, while data regarding long-term clinical outcomes in primary teeth are scarce [14,15]. In particular, prospective studies with extended follow-up periods evaluating biological, anatomical, esthetic, and functional criteria using standardized assessment systems are lacking.

In light of these considerations, the aim of the present study was to prospectively evaluate the clinical effectiveness of chemo-mechanical caries excavation using Brix 3000 in primary teeth over an 18-month follow-up period. The findings of this study provide clinically relevant evidence regarding the long-term performance, safety, and applicability of this minimally invasive approach in pediatric dental practice.

## 2. Materials and Methods

### 2.1. Study Design and Ethical Considerations

This clinical study was conducted in accordance with the principles of the Declaration of Helsinki. Ethical approval was obtained from the Ethics Committee of the Medical University—Sofia (Protocol No. KENIMUS 05/20, dated 20 February 2019). Written informed consent was obtained from the parents or legal guardians of all participating children prior to their inclusion in the study.

### 2.2. Study Population and Eligibility Criteria

Study Population

A total of 82 children aged 4–7 years were enrolled in the study, each presenting with at least one deep dentin carious lesion in a primary molar (ICDAS II code 05/06).

Inclusion Criteria:

- Proximal or occlusal carious lesion of a first or second primary molar;
- Absence of clinical symptoms, including spontaneous, nocturnal, or periodontal pain;
- Absence of periapical change;
- Expected time to physiological tooth exfoliation of at least 18 months;
- Radiographic evidence of a thin layer of demineralized dentin covering the pulp horn.

Exclusion Criteria:

- Presence of clinical symptoms (spontaneous, nocturnal, or periodontal pain);
- Presence of periapical changes;
- Less than 18 months until physiological tooth exfoliation.

Grouping According to Excavation Method

The carious lesions were grouped according to the method of excavation:

- Group 1 (control)—40 lesions treated using conventional bur excavation;
- Group 2—42 lesions treated using chemo-mechanical excavation with Brix 3000.

### 2.3. Treatment Protocol and Dentin Assessment

The carious lesions were treated according to the depth and characteristics of the affected dentin using two distinct protocols: conventional bur excavation (Group 1, control) and chemo-mechanical excavation with Brix 3000 (Group 2).

Group 1—Conventional Bur Excavation

Cavity preparation involved the removal of carious dentin from the enamel–dentin junction and cavity walls, followed by selective removal from the cavity floor depending on lesion depth. Deep dentin lesions (ICDAS code 05) were excavated to affected dentin and lined with a calcium hydroxide liner (Biner LC, Meta Biomed Co., Ltd., Cheongju, Republic of Korea) before restoration with compomer (Glasiosite, VOCO, Cuxhaven, Germany). For cases classified as asymptomatic closed pulpitis (ICDAS code 06), indirect pulp capping was performed. In these cases, partially infected dentin was left in situ, a calcium hydroxide liner (caviLINE, VOCO, Cuxhaven, Germany) was applied, and the cavity was restored with the same compomer.

Group 2—Chemo-Mechanical Excavation with Brix 3000

The chemo-mechanical protocol followed the same restorative principles. Brix 3000 was applied to the cavity and allowed to act for 2 min, as recommended by the manufacturer. Carious dentin was then removed using a curved excavator (Koine, Koine Italia snc, Milan, Italy). Restoration was performed in an identical manner to the procedure used for Group 1.

**Figure 1:**
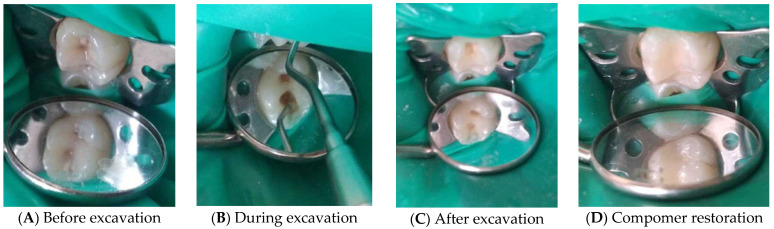
Clinical case of chemo-mechanical caries removal using Brix 3000 in a 5-year-old child. (**A**) Initial clinical appearance showing a cavitated carious lesion corresponding to ICDAS code 05, with visible dentin involvement. (**B**) Application of the chemo-mechanical agent Brix 3000 followed by gentle excavation of infected dentin using a hand excavator. (**C**) Post-excavation view demonstrating the presence of affected dentin, indicating selective caries removal and preservation of remineralizable tissue. (**D**) Final restoration with a compomer material, ensuring adequate cavity sealing and anatomical reconstruction of the tooth.

Figure 1 demonstrates the sequential steps of chemo-mechanical caries excavation in a 5-year-old patient, including the initial carious lesion, application of Brix 3000, excavation of infected dentin, and final compomer restoration.

Dentin Assessment and Fluorescence Control

During each stage of excavation, the remaining dentin was evaluated using a fluorescence-based device (ProFace, W&H Dentalwerk Bürmoos GmbH, Bürmoos, Austria). The method employed violet light (λ ≤ 405 nm) and protective eyewear with a filter passing light with a wavelength of up to 500 nm. Fluorescent criteria were followed to guide the decision on the removal of dentin.

- Infected dentin: intense red fluorescence—requires removal.
- Partially infected dentin: pink fluorescence with localized red spots in the sub-pulpal dentin.
- Affected dentin: pale pink fluorescence limited to isolated areas at the cavity floor.
- Healthy dentin: absence of fluorescence.

This approach ensured standardized removal of carious dentin while preserving affected or healthy tissue and minimized unnecessary pulp exposure. The fluorescence-guided dentin assessment was integral to both conventional and chemo-mechanical protocols and formed the basis for subsequent clinical evaluations.

**Figure 2:**
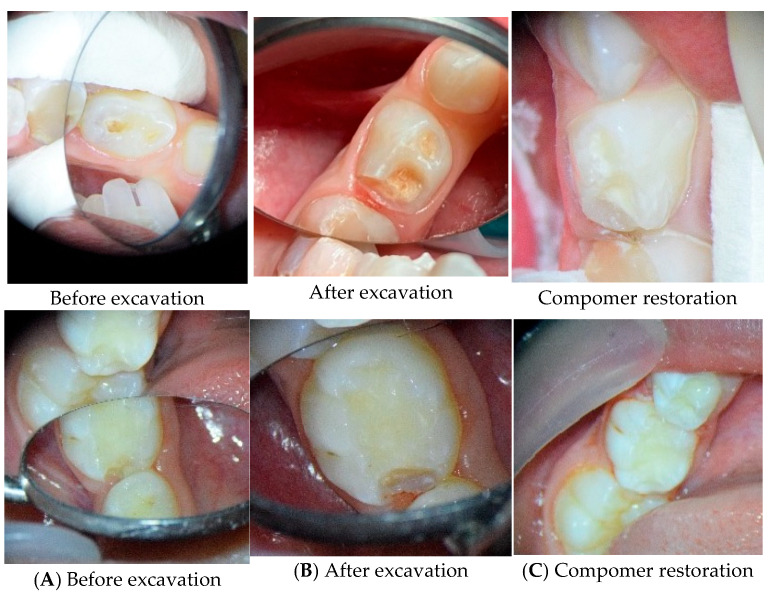
Additional clinical cases of chemo-mechanical caries removal using Brix 3000. (**A**) Initial presentation of the carious lesion before excavation. (**B**) Post-excavation view showing affected dentin preserved for remineralization. (**C**) Final compomer restoration, demonstrating proper anatomical reconstruction and cavity sealing.

Figure 2 shows additional clinical cases illustrating the same treatment protocol, highlighting the before-and-after stages of dentin removal and restoration.

### 2.4. Prospective Follow-Up and Clinical Criteria

A prospective follow-up protocol was established to monitor the treated lesions over an 18-month period. Follow-up examinations were performed at 1 week and 1, 3, 6, 12, and 18 months after treatment. Each visit included a clinical assessment of the restoration and the treated tooth, as well as radiographic evaluation when necessary.

Clinical evaluation was based on an adaptation of the FDI (Fédération Dentaire Internationale) criteria for direct and indirect restorations, with particular emphasis on biological outcomes [20]. For the purpose of this study, the FDI criteria were simplified in the esthetic, anatomical, and functional evaluation domains, while biological criteria were preserved and expanded to capture key aspects relevant to deep dentin lesions (for detailed scoring codes, see Table S1 in the Supplementary Materials).

Biological Criteria:

- Postoperative sensitivity: graded from no sensitivity to prolonged sensitivity (>1 week but <6 months).
- Acute symptomatology and complications: including pulp-related symptoms, soft tissue inflammation, abscess formation, or need for re-intervention.

Esthetic Criteria:

- Surface gloss and roughness: compared to adjacent enamel, graded from similar to markedly different.
- Marginal discoloration: ranging from absent to unacceptable discoloration requiring restoration replacement.

Anatomical Criteria:

- Restoration contour and form: graded from fully matching tooth anatomy to poor anatomical match.

Functional Criteria:

- Integrity and marginal adaptation: graded from no defects to complete loss of restoration or compromised proximal contacts.

This standardized follow-up protocol allowed for objective assessments of the clinical and biological performance of conventional and chemo-mechanical excavation methods, enabling a comparison of outcomes between treatment groups and over time.

The study was conducted as a prospective clinical observational study. Allocation to treatment groups was not randomized and was based on clinical decision-making at the time of treatment.

All clinical procedures were performed by a single dentist who was a certified specialist in pediatric dentistry, with experience in minimally invasive treatment approaches.

All follow-up clinical evaluations were performed by the same experienced clinician to ensure the consistency of the assessments. However, a formal calibration procedure was not conducted prior to the study. Due to the nature of the interventions, blinding was not feasible.

A formal sample size calculation was not performed prior to the initiation of the study. The sample size was determined based on the number of eligible clinical cases treated during the study period.

### 2.5. Statistical Analysis

Statistical analysis was performed using IBM SPSS Statistics software (version 19.0) and Microsoft Excel 2019. Descriptive statistics were used to summarize the data, including absolute and relative frequencies (number and percentage) for categorical variables.

Comparisons between the two treatment groups were conducted using Fisher’s exact test (two-sided) for categorical variables. Fisher’s exact test was selected due to the presence of small expected cell counts in some comparisons. The *p*-values reported in the tables correspond to the exact two-sided significance values.

The unit of analysis was the treated carious lesion.

A *p*-value < 0.05 was considered to indicate statistical significance.

## 3. Results

The clinical outcomes of the treated lesions were monitored throughout the 18-month follow-up period. Table 1 summarizes the number of lesions observed at each follow-up interval, according to the type of cavity preparation. Cases lost due to physiological exfoliation or complications are indicated in parentheses (*, **). A total of 82 carious lesions were initially assessed for eligibility. Of these, 73 lesions met the inclusion criteria and were included in the prospective clinical follow-up at baseline. The remaining nine cases were excluded due to non-attendance at the initial evaluation.

By the end of the follow-up period, a total of 66 carious lesions were monitored, including 31 lesions treated with conventional bur excavation and 35 lesions treated with chemo-mechanical excavation using Brix 3000. The primary focus of the results was on biological criteria as the main indicator of clinical success in primary teeth. Restoration quality was summarized as a secondary outcome.

**Figure 3:**
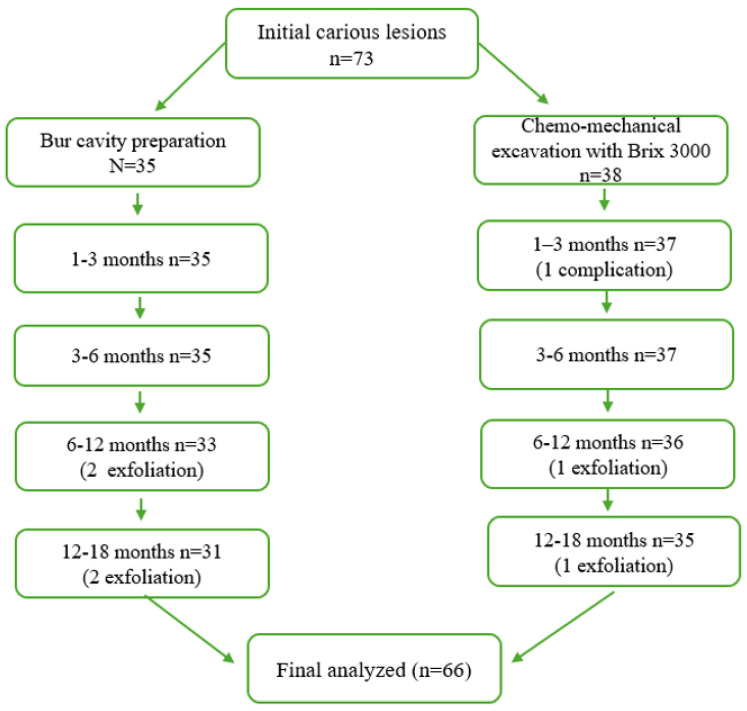
Flow diagram illustrating case allocation, follow-up, and losses to follow-up during the 18-month study period.

The decrease in the number of cases during follow-up was due to physiological exfoliation of primary teeth and a single case requiring intervention. A flow diagram illustrating the case allocation, follow-up, and losses to follow-up during the 18-month study period is presented in Figure 3.

Table 2 and Table 3 present the number and percentage of lesions without postoperative sensitivity and without acute symptoms, respectively, at each follow-up interval. The unit of analysis is the treated lesion, and losses are accounted for as described in Table 1.

It is noteworthy that postoperative sensitivity was reported in only one case during the first week after completion of the treatment, and this case was recorded in the control group in which cavity preparation was performed using conventional bur excavation. This case was categorized as code 1, indicating the presence of short-term postoperative sensitivity. However, the tooth was not excluded from further follow-up as the postoperative sensitivity resolved spontaneously and no subsequent complications were observed.

During the final follow-up period (12–18 months), the decrease in the number of cases was attributed to the physiological exfoliation of primary teeth.

At one month after the procedure, one case treated with Brix 3000 presented with exacerbation and acute periodontal symptomatology requiring endodontic treatment. Consequently, this case was excluded from further follow-up. No additional complications were recorded in the remaining cases during the subsequent follow-up periods (3–6, 6–12, and 12–18 months).

The decrease in the number of analyzed cases toward the end of the observation period, regardless of the treatment method used, was attributed to the physiological exfoliation of primary teeth and was not related to treatment failure.

The overall follow-up results are summarized in Table 4, which presents the number and relative proportion of clinical successes and failures according to the monitored criteria for each treatment group and follow-up interval. Biological criteria were considered the primary and most clinically relevant outcomes for evaluating treatment success, whereas the remaining clinical parameters were assessed as secondary outcomes.

The applied treatment protocols were observed to be highly effective in terms of the biological criteria throughout the follow-up period, with only a single case presenting acute periodontal symptoms that required intervention.

Assessment of esthetic criteria revealed that approximately one-third of restorations exhibited minor deviations, mainly observed in later follow-up periods (6–12 and 12–18 months). These changes are likely related to the characteristics of the compomer material used, which is recommended for use in primary teeth with expected physiological exfoliation. Over time, compomer surfaces may become slightly rougher due to ion loss from the glass phase, especially under lower pH conditions [21].

The anatomical and functional criteria showed only minor deviations, which were observed mainly during the later follow-up periods (6–12 and 12–18 months). These deviations affected a limited number of restorations and did not compromise their clinical acceptability. The observed changes are most likely related to the limited wear resistance of the compomer material used; however, all restorations remained functional and clinically acceptable throughout the follow-up period.

A comparative analysis between conventional bur excavation and chemo-mechanical excavation with Brix 3000 was performed for all evaluated criteria over the entire 18-month follow-up period. The distribution of successful and unsuccessful outcomes for each treatment method is presented in Table 5.

Fisher’s exact test (two-sided) was used for comparisons between groups. Unit of analysis: treated lesion.

No statistically significant differences were observed between the two excavation methods for any of the monitored criteria throughout the follow-up period (*p* > 0.05). These results indicate that chemo-mechanical excavation with Brix 3000 provides clinical outcomes comparable to those achieved with conventional bur excavation in the treatment of deep carious lesions in primary teeth.

**Figure 4:**
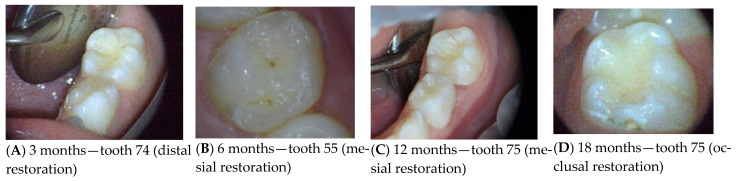
Representative clinical outcomes of restorations at different follow-up intervals after chemo-mechanical caries removal using Brix 3000. (**A**) Three-month follow-up of tooth 74 (distal restoration), showing good marginal integrity and preserved anatomical contour. (**B**) Six-month follow-up of tooth 55 (mesial restoration), demonstrating stable restoration with no marginal discoloration or structural deterioration. (**C**) Twelve-month follow-up of tooth 75 (mesial restoration), indicating maintained surface smoothness, anatomical morphology, and functional stability. (**D**) Eighteen-month follow-up of tooth 75 (occlusal restoration), showing long-term clinical success with intact margins, proper occlusal anatomy, and no evidence of restoration failure.

Figure 4 illustrates representative clinical outcomes at 3, 6, 12, and 18 months after chemo-mechanical caries removal, showing the esthetic, anatomical, and functional performance of restorations over time.

## 4. Discussion

This prospective clinical study aimed to evaluate and compare the clinical outcomes of primary teeth treated with conventional bur excavation and chemo-mechanical excavation using Brix 3000. For this purpose, the FDI criteria for the evaluation of direct and indirect restorations were applied and adapted to the specific objectives of the study. Particular emphasis was placed on biological criteria, as they represent the most clinically relevant indicators of treatment success in primary teeth and directly reflect pulp status and the occurrence of postoperative complications.

The clinical and biological outcomes observed in the present study suggest a high level of clinical effectiveness of both conventional bur excavation and chemo-mechanical excavation with Brix 3000 in the treatment of carious lesions classified as ICDAS II codes 05/06 in primary molars. Given the minimal differences in success rates across the biological, esthetic, anatomical, and functional criteria, in addition to the absence of statistically significant differences between the two treatment methods, these findings provide evidence supporting the discussion of the clinical relevance and potential advantages of chemo-mechanical excavation in pediatric dentistry.

From a clinical perspective, the results obtained support the use of chemo-mechanical excavation with Brix 3000 as an appropriate treatment approach in pediatric dentistry, particularly in children with increased dental anxiety, limited cooperation, or in situations where conventional bur excavation may be difficult to perform. The ability to preserve pulp vitality without increasing the risk of postoperative complications represents a substantial advantage of this method and aligns with the principles of minimally invasive dentistry.

The results of the present study indicate that the factors characterizing the biological effectiveness of treatment with Brix 3000 and the applied controlled excavation protocol are associated with a high success rate approaching 100%. Only one case treated with Brix 3000 demonstrated spontaneous periodontal pain one month after treatment. This case involved a first primary maxillary molar and may be explained by the anatomical characteristics of the tooth, including a highly projected pulp horn, as well as the limited ability of the pulp tissue to resolve inflammation.

Nevertheless, several methodological aspects should be considered when interpreting these findings. The relatively small sample size, particularly at the final follow-up interval, may limit the generalizability of the results. The decrease in the number of monitored cases over time, primarily due to physiological exfoliation of primary teeth, further reduced the available sample for long-term analysis.

In addition, group allocation was not randomized, which may introduce potential selection bias. All clinical evaluations were performed by a single examiner without formal calibration, and blinding was not feasible due to the nature of the interventions. These factors may have influenced the outcome assessments.

Although appropriate statistical tests for categorical data were applied, the limited sample size and the distribution of certain outcomes restricted the scope of inferential statistical analysis. Therefore, the results should be interpreted with caution.

Al-Zayer suggests that first primary maxillary molars may be more prone to complications following indirect pulp treatment compared with second primary molars, although this tendency was not statistically confirmed [22].

The anatomical and functional criteria evaluated in the present study demonstrated success rates exceeding 95%, which represents a strong indicator of overall treatment effectiveness. In contrast, minor deviations were observed with respect to the esthetic properties of the restorations. During the follow-up period, approximately one-third of the evaluated cases exhibited some degree of esthetic change.

These changes were not associated with the chemo-mechanical excavation agent or the applied excavation protocol, and are more likely due to the properties of the compomer material used for restoration. The selected compomer is recommended for primary teeth with expected physiological exfoliation, where long-term esthetic stability is of secondary importance compared with biological safety and functional integrity.

The fact that the observed deviations in the esthetic, anatomical, and functional criteria predominantly occurred during the later follow-up periods supports the assumption that these changes are more closely related to the characteristics of the restorative material rather than to the method of cavity preparation. This finding suggests that the excavation technique itself does not negatively influence the long-term clinical performance of the treated teeth.

The results of the present study found no statistically significant differences between the two excavation methods—conventional bur excavation and chemo-mechanical excavation with Brix 3000. Chemo-mechanical excavation did not differ from conventional preparation with respect to biological, esthetic, anatomical, or functional outcomes.

Numerous studies in the literature have evaluated and compared the clinical success of conventional bur excavation and enzymatic caries removal techniques in primary teeth over extended follow-up periods. Motta et al. investigated the effectiveness of enzymatic excavation using Papacárie^®^, the original formulation preceding Brix 3000, in comparison with conventional bur excavation in primary teeth over an 18-month period, using both clinical and radiographic evaluations. The authors reported no radiographic differences between the two excavation methods and concluded that enzymatic caries removal can be successfully applied in pediatric patients [23]. These findings demonstrate the high clinical effectiveness of enzymatic caries excavation and are consistent with the results of the present study.

Another study followed 84 primary teeth treated with Papacárie^®^ over a 12-month period. The authors assessed the radiographic findings and restoration quality and reported that 90% of the restorations were classified as satisfactory and successful, while only 10% were considered unsatisfactory. The authors likewise concluded that enzymatic caries excavation is an effective and successful approach for the long-term treatment of carious lesions in primary teeth [24].

Phonghanyudh et al. evaluated a total of 276 primary molars by comparing excavation limited to the enamel–dentin junction following ART (Atraumatic Restorative Treatment) principles with conventional bur excavation. The restorations were assessed over a 12-month period, and success rates exceeding 80% were reported for all three groups. Only two cases of fistula formation were observed, both in the conventionally prepared group, and only one child reported discomfort during mastication in the group treated with excavation limited to the enamel–dentin junction [25]. Another clinical investigation assessed the success of chemo-mechanical caries removal with Papacárie in primary teeth over a 12-month follow-up period. The findings indicate that enzymatic excavation was associated with a high clinical success rate, supporting its effectiveness as a minimally invasive treatment approach in pediatric patients [26]. All cited studies support our findings that chemo-mechanical caries removal using Brix 3000 demonstrates a clinical success rate comparable to that of conventional rotary excavation.

In addition to studies evaluating papain-based agents in general, several clinical investigations have specifically assessed the performance of Brix 3000. These studies report comparable caries removal efficacy to conventional rotary excavation, reduced patient discomfort, and favorable microbiological outcomes. However, most of these investigations focused on short-term results and did not include extended prospective follow-up periods [14,27,28].

Although several studies have investigated papain-based chemo-mechanical caries removal agents, long-term prospective clinical studies specifically evaluating Brix 3000 are scarce. Most available publications focused on short-term clinical outcomes, handling properties, or patient comfort. To the best of our knowledge, extended follow-up data assessing the biological, anatomical, esthetic, and functional outcomes of Brix 3000 in primary teeth remain limited. Therefore, the present study contributes novel clinical evidence regarding its long-term performance over an 18-month follow-up period.

No statistically significant differences were observed between conventional bur excavation and chemo-mechanical excavation with Brix 3000 with respect to biological, esthetic, anatomical, or functional outcomes. These findings suggest that chemo-mechanical excavation may represent a clinically comparable alternative to conventional rotary preparation in the treatment of carious lesions in primary teeth.

However, given the non-randomized design, limited sample size, and decrease in cases during the long-term follow-up, these results should be interpreted with caution. Further randomized controlled clinical trials with larger sample sizes and extended follow-up periods are required to confirm the present findings.

Despite these limitations, the findings provide valuable long-term clinical data on Brix 3000, for which extended prospective evidence remains limited.

## 5. Limitations

The present study has several limitations. The number of cases decreased over time due to physiological exfoliation of primary teeth, resulting in a smaller sample size at the final follow-up. Group allocation was not randomized, and all clinical evaluations were performed by a single clinician without formal calibration. Blinding was not feasible due to the nature of the interventions. In addition, no formal sample size calculation was performed, and the study was not prospectively registered in a clinical trial registry. Despite these limitations, the prospective design and systematic follow-up provided meaningful clinical data.

## 6. Conclusions

The results of this 18-month prospective clinical study demonstrate a high level of clinical success for chemo-mechanical caries excavation with Brix 3000 in the treatment of carious lesions in primary teeth. The method exhibited excellent biological outcomes and clinically acceptable esthetic, anatomical, and functional performance throughout the follow-up period.

The absence of statistically significant differences between chemo-mechanical excavation with Brix 3000 and conventional bur excavation indicates that the enzymatic approach represents a clinically comparable and reliable alternative for cavity preparation in the primary dentition. Given its minimally invasive nature and favorable biological performance, chemo-mechanical excavation with Brix 3000 could be considered a suitable option in pediatric dentistry, particularly in situations where reductions in discomfort, anxiety, and the need for local anesthesia are desired.

## Figures and Tables

**Table 1:** Number of clinical cases during follow-up.

	Follow-Up	Start	1–3 mo.	3–6 mo.	6–12 mo.	12–18 mo.
Period Cavity Preparation Method						
Conventional bur excavation		35	35	35	33 (−2 **)	31 (−2 **)
Chemo-mechanical excavation with Brix 3000		38	37 (−1 *)	37	36 (−1 *)	35 (−1 **)
Teeth remaining at follow-up		73	72	72	69	66

Notes: * Tooth lost due to complication; ** tooth lost due to physiological exfoliation.

**Table 2:** Number of cases without postoperative sensitivity during the 18-month follow-up period.

Period	Successes				Failures				Fisher’s Exact Test
	Bur		Brix 3000		Bur		Brix 3000		
	N	%	N	%	N	%	N	%	
1 week	34	97.4%	38	100%	1	2.6%	-	-	*p* = 1.000
1–3 months	34	100%	37	100%	-	-	-	-	
3–6 months	33	100%	37	100%	-	-	-	-	
6–12 months	33	100%	36	100%	-	-	-	-	
12–18 months	31	100%	35	100%	-	-	-	-	

**Table 3:** Number of cases without acute symptomatology throughout the follow-up period.

Period	Successes				Failures				Fisher’s Exact Test
	Bur		Brix 3000		Bur		Brix 3000		
	N	%	N	%	N	%	N	%	
1–3 months	35	100%	36	97.4%	-	-	1	2.6%	*p* = 1.000
3–6 months	33	100%	37	100%	-	-	-	-	
6–12 months	33	100%	36	100%	-	-	-	-	
12–18 months	31	100%	35	100%	-	-	-	-	

**Table 4:** Relative proportion of successes and failures in the monitored clinical cases.

Criteria	Follow-Up Period	Successes	Failures	Total
		N (%)	N; %	N; %
Biological	1–3 mo.	72 (98.6%)	1 (1.4%)	73 (100%)
	3–6 mo.	72 (100%)	0 (0%)	72 (100%)
	6–12 mo.	69 (100%)	0 (0%)	69 (100%)
	12–18 mo.	66 (100%)	0 (0%)	66 (100%)
Esthetic	3–6 mo.	72 (100%)	0 (0%)	72 (100%)
	6–12 mo.	51 (73%)	18 (27%)	69 (100%)
	12–18 mo.	43 (65%)	23 (35%)	66 (100%)
Anatomical	3–6 mo.	72 (100%)	0 (0%)	72 (100%)
	6–12 mo.	65 (94%)	4 (6%)	69 (100%)
	12–18 mo.	63 (95%)	3 (5%)	66 (100%)
Functional	3–6 mo.	72 (100%)	0 (0%)	72 (100%)
	6–12 mo.	66 (96%)	3 (4%)	69 (100%)
	12–18 mo.	65 (98%)	1 (2%)	66 (100%)

**Table 5:** Comparison of clinical outcomes between conventional bur excavation and chemo-mechanical excavation with Brix 3000 over the 18-month follow-up period.

Criteria	Successes				Failures				Fisher’s Exact Test
	Bur		Brix 3000		Bur		Brix 3000		
	N	%	N	%	N	%	N	%	
Biological	31	100%	34	97.1%	-	-	1	2.9%	*p* = 1.000
Esthetic	19	61.3%	24	68.6%	12	38.7%	11	31.4%	*p* = 0.609
Anatomical	29	93.5%	34	97.1%	2	6.5%	1	2.9%	*p* = 0.597
Functional	31	100%	34	97.1%	0	0%	1	2.9%	*p* = 1.000

## Data Availability

The original contributions presented in the study are included in the article; further inquiries can be directed to the corresponding author.

## Supplementary Materials

The following supporting information can be downloaded at https://www.mdpi.com/article/10.3390/medicina62040615/s1: Table S1. Detailed scoring codes for biological, esthetic, anatomical, and functional criteria used in the prospective follow-up of primary molar restorations.

## Abbreviations

The following abbreviations are used in this manuscript:

ICDAS II	International Caries Detection and Assessment System
FDI	Fédération Dentaire Internationale
ART	Atraumatic Restorative Treatment
