# Supplementary material for: Clinical and Biological Evaluation of Chemo-Mechanical Caries Excavation with Brix 3000 in Primary Molars: An 18-Month Prospective Study
Source: Medicina (Kaunas). 2026 Mar 24;62(4):615. doi: 10.3390/medicina62040615 (PMC13117672; doi:10.3390/medicina62040615)
Supplement: Supplementary file 1 [file medicina-62-00615-s001.zip › medicina-4182475-supplementary.pdf]

Table S1. Detailed clinical scoring codes for follow-up of primary molar restorations.

Domain	Subdomain	Code	Description
**Biological criteria**	Postoperative sensitivity	0	No postoperative sensitivity
		1	Brief postoperative sensitivity
		2	Prolonged sensitivity (>1 week but <6 months); complaints from cold stimuli, normal pulp reaction
		A	Physiological exfoliation
	Acute symptomatology and complications	0	Absence of acute symptomatology
		1	Acute postoperative sensitivity requiring re-intervention
		2	Swelling, redness, fistula, or abscess in apex/soft tissues
		3	No sensitivity and pulp communication
		A	Physiological exfoliation
**Aesthetic criteria**	Surface gloss and roughness	1	Approximately same as adjacent enamel
		2	Different from enamel within acceptable limits
		3	Significantly different from enamel
		A	Physiological exfoliation
	Marginal discoloration	1	Absent
		2	Moderate marginal discoloration
		3	Significant discoloration
		4	Unacceptable discoloration requiring restoration replacement
		A	Physiological exfoliation

Domain	Subdomain	Code	Description
**Anatomical criteria**	Restoration contour and form	1	Anatomical form fully matching tooth
		2	Anatomical form partially matching tooth
		3	Anatomical form not matching tooth
		A	Physiological exfoliation
**Functional criteria**	Integrity and marginal adaptation	1	No fractures or cracks
		2	Several cracks, but no compromised marginal adaptation or proximal contact
		3	Cracks affecting restoration quality and/or proximal contact; partial restoration loss
		4	Total restoration loss
		A	Physiological exfoliation
